# Oven-Controlled MEMS Oscillator with Integrated Micro-Evaporation Trimming

**DOI:** 10.3390/s20082373

**Published:** 2020-04-22

**Authors:** Binbin Pei, Ke Sun, Heng Yang, Chaozhan Ye, Peng Zhong, Tingting Yu, Xinxin Li

**Affiliations:** 1 State Key Laboratory of Transducer Technology, Shanghai Institute of Microsystem and Information Technology, Chinese Academy of Sciences, Shanghai 200050, China; binbinp@mail.sim.ac.cn (B.P.);; 2 School of Graduate Study, University of Chinese Academy of Sciences, Beijing 100049, China

**Keywords:** frequency trimming, integrated micro-evaporation trimming, oven-controlled MEMS oscillator

## Abstract

This study reports an oven-controlled microelectromechanical systems oscillator with integrated micro-evaporation trimming that achieves frequency stability over the industrial temperature range and permanent frequency trimming after vacuum packaging. The length-extensional-mode resonator is micro-oven controlled and doped degenerately with phosphorous to achieve a frequency instability of ±2.6 parts per million (ppm) in a temperature range of −40 to 85 °C. The micro-evaporators are bonded to the resonator, integrated face-to-face, and encapsulated in vacuum. During trimming, the micro-evaporators are heated electrically, and the aluminum layers on their surfaces are evaporated and deposited on the surface of the resonator that trims the resonant frequency of the resonator permanently. The impact of the frequency trimming on the temperature stability is very small. The temperature drift increases from ±2.6 ppm within the industrial temperature range before trimming to ±3.3 ppm after a permanent trimming of −426 ppm based on the local evaporation of Al. The trimming rate can be controlled by electric power. A resonator is coarse-trimmed by approximately −807 ppm with an evaporation power of 960 mW for 0.5 h, and fine-trimmed by approximately −815 ppm with an evaporation power of 456 mW for 1 h. Though the *Q*-factor decreases after trimming, a *Q*-factor of 304,240 is achieved after the trimming of −1442 ppm.

## 1. Introduction

Microelectromechanical systems (MEMS) oscillators have been extensively researched as replacements for quartz crystal oscillators in timing and communication applications [1,2,3]. Compared with quartz crystal oscillators, MEMS oscillators are small-scale, low cost, and allow for high-volume manufacturing and integration. 

Oscillators must achieve frequency accuracy as well as temperature stability at the ppm level in the applications of frequency reference sources. Frequency trimming is required for frequency accuracy because frequency accuracy is limited by the tolerances of the MEMS processes, such as deposition, lithography, and etching, that leads to frequency deviations spanning several hundred ppm, even with design for manufacturability (DFM) [4,5]. In addition, temperature compensation is required for temperature stability because the temperature coefficient of the resonant frequency (TCF) of silicon is up to −30 ppm/°C, which is more than 100 times larger than that of quartz crystal oscillators. It was demonstrated that frequency accuracy at the ppm level and temperature stability can be achieved with frequency synthesizer techniques, the drawback of which is the relatively high power. The power of the frequency synthesized MEMS oscillator is approximately 10 times larger than those of quartz crystal oscillators of the same level owing to the increased powers of the phase-locked loop (PLL) circuits, and limits its applications [6,7,8,9,10,11,12]. Low-power techniques are required for both frequency tuning and temperature stabilization. 

It is really a challenge to trim the MEMS resonators because the MEMS resonators must be vacuum-packaged before measurements and trimmed after packaging. The traditional metal deposition trimming technique verified in quartz crystal trimming is difficult to implement for frequency trimming after vacuum packaging. Post-package pulsed-laser-deposition frequency trimming technologies have been demonstrated for MEMS resonators [5]. However, the resonator frequency has not yet reached the ppm level accuracy owing to the difficulty of controlling the geometry and the amount of the materials deposited by laser. Another laser trimming method was based on the material removal trimmed resonator from an initial frequency error to 21 ppm [13,14]. However, the resonators were not packaged so that the resonator frequency would shift after the packaging. Furthermore, laser frequency trimming technologies can only be employed by the oscillators with transparent packages. Samarao et al. proposed a frequency trimming method based on electric heating that changed the equivalent stiffness by heating the metal into the resonator at the cost of *Q*-factor reductions [15]. Integrated micro-evaporation trimming (IMET) has been proposed by the authors to achieve coarse and fine frequency tuning with Al evaporation by integrated micro-evaporators [16]. The permanent frequency trimming can be performed during the testing processes of the oscillators after vacuum packaging, which is promising for the low-power MEMS oscillators.

Low-power temperature stability at the ppm level can also be achieved with the use of micro-oven control combined with degenerate phosphorous doping [17,18,19,20,21,22,23]. It has been demonstrated that the TCF of the [100]-oriented silicon resonators is nonlinear, and there is a turnover temperature point [24,25] at which the TCF is equal to zero. The turnover point can be tuned by the doping level [26]. The oven-controlled N^++^ [100] length-extensional mode silicon resonator has been previously reported by the authors [21,22,23]. Owing to the very low TCF near the turnover temperature point of the N^++^ structure, a lookup table-based control algorithm was sufficiently accurate to achieve temperature stability at the ppm level from −40 to 85 °C. 

This study introduces a MEMS oscillator with IMET technology for frequency trimming and micro-oven-control for temperature stabilization. The oven-controlled N^++^ [100] silicon resonator in [21,22] is adopted in this investigation. Evaporation trimming is used in this study to adjust the resonant frequency of the oven-controlled MEMS oscillator (OCMO). Before trimming, the frequency stability is less than ±2.6 ppm over the entire temperature range, and it increase to ±3.3 ppm after a permanent trimming of −426 ppm. This study demonstrates that the effect of evaporation trimming on the frequency temperature stability of the OCMO over the entire temperature range is less than 1.5 ppm after a frequency trimming of −426 ppm.

## 2. Principle and Design

### 2.1. Principle of the IMET

Because the masses of MEMS resonators are extremely small, the resonant frequencies can be trimmed significantly with trace amounts of material deposition. When a small amount of material, Δ*m*, is deposited on the resonator, the resonant frequency is trimmed down, and Δ*f_0_* is determined using the following equation:

(1) $$\frac{\Delta f_{0}}{f_{0}}=-\frac{\Delta m}{2m_{eff}}$$

where *m_eff_* is the effective mass of the resonator, which was as low as 1.84 × 10^−8^ g in this study. Theoretically, a permanent frequency trimming of approximately −1000 ppm can be achieved with 36.8 pg of material deposited on the I^2^-BAR resonator used in this study. 

Owing to the small thermal capacity, the suspended MEMS structures, such as beam-mass structures, can be locally heated to reach several hundred degrees Centigrade with electrical heating in vacuum. Small amounts of the metal material on top of the suspended structures can be evaporated to trim the oscillator permanently. The evaporation can be estimated with the Hertz–Knudsen formula owing to its simple form, particularly in practical application cases. The mass flux of pure metal can be approximated as follows [27]:

(2) $$E=\alpha p\sqrt{\frac{M}{2\pi RT_{eva}}}$$

where *α* is the evaporation coefficient which has a value equal to one in the ideal evaporation state when all the metal evaporated from the surface of the metal source condenses on the resonator. *M* and *p* are the molecular weight and standard vapor pressure of the evaporating material, *R* is the universal gas constant, and *T_eva_* is the temperature at the evaporating surface. According to the Equation (2), the materials with high vapor pressure at a relatively low temperature are preferred. Aluminum is selected in this design, because it has good adhesion to silicon and the standard vapor pressure of Al is ~6.44 × 10^−4^ Pa at 850 °C and ~2.6 × 10^−3^ Pa at 900 °C [28]. Estimation based on Equation (2), the mass fluxes of pure aluminum are 4.37 × 10^−7^ kg/(m^2^·s) and 1.71 × 10^−6^ kg/(m^2^·s) at 850 and 900 °C, respectively. The standard vapor pressure of pure aluminum Al can be approximated as follows [29]:

(3) $$\mathrm{lg}p_{Al}=-\frac{16380}{T_{eva}}+14.445-\mathrm{lg}T_{eva}$$

**Figure 1:**
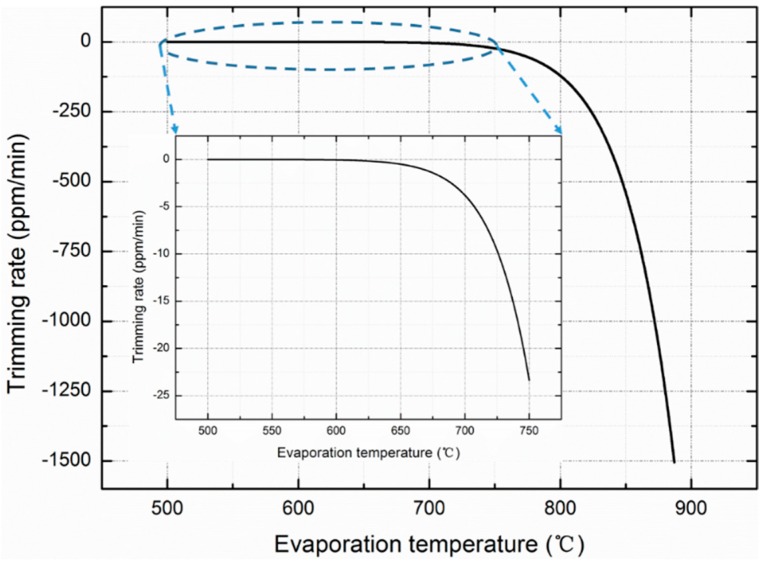
Relationship between evaporation temperature and rate constructed theoretically in the evaporation ideal state according to Equations (1)–(3).

According to Equations (1)–(3), the relationship between the evaporation temperature and rate is built theoretically in the ideal evaporation state, as shown in Figure 1. When the size of the aluminum source is 20 μm × 38 μm, as used in this study, the trimming rate of the resonant frequency is estimated to be approximately −541 ppm/min in the ideal evaporation state when the aluminum source is heated to 850 °C. It takes approximately 2 min to trim more than −1000 ppm that can be achieved during the testing processes of the oscillators after vacuum packaging.

Two evaporator boats supported by two silicon heating beams are employed to heat the evaporating material. During operation, electric power is applied to the heating beams, and the evaporator boat is heated by Joule heating at the evaporation temperature. At this temperature, the aluminum atoms escape from the source material and reach the micromechanical resonator surface. When the distance between the evaporator boat and the resonator after bonding (about 40 μm) is less the mean free path of aluminum (about 176 μm when aluminum is heated to 866 °C in 500 Pa vacuum chamber), the collision of aluminum atoms with the residual molecules in the vacuum chamber can be ignored. According to Fourier’s law, the temperature of the evaporator boat can be approximated as follows,

(4) $$T_{eva}=\frac{PL}{2khb}+T_{a}$$

where *P* is the Joule heating power for evaporation, *k* is thermal conductivity of a single crystal silicon, *T_a_* is the ambient temperature, and *L*, *b*, and *h*, are the length, width, and thickness of the heating beam, respectively. When a heating power of 108 mW is applied, the evaporator boat can be heated to 854 °C based on Equation (4).

### 2.2. Design of the OCMO with IMET

**Figure 2:**
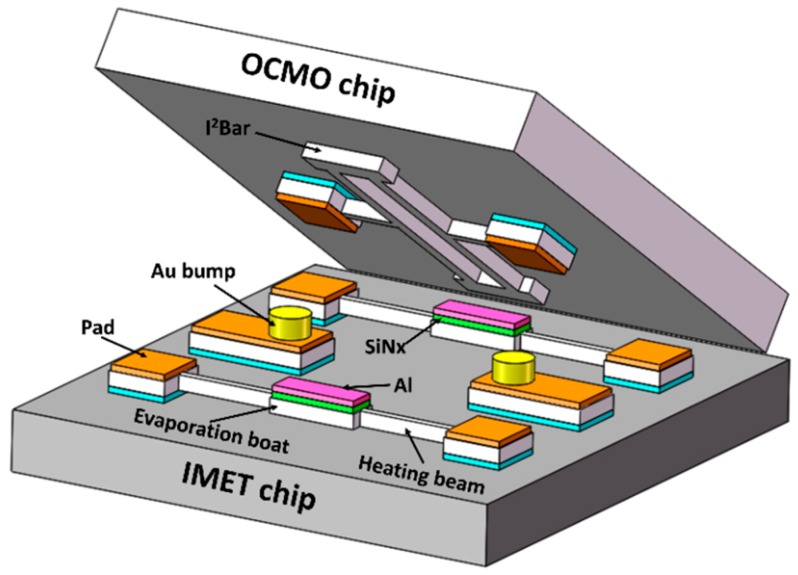
Schematic view of the integrated micro-evaporation trimming (IMET) chip directly bonded to the oven-controlled microelectromechanical system (MEMS) oscillator (OCMO) chip face-to-face.

The structure of the OCMO with IMET is shown in Figure 2. The IMET chip is bonded to the OCMO chip face-to-face. 

The [100] length-extensional (LE) mode silicon resonator was doped degenerately with phosphorous to tune the turnover temperature to approximately 110 °C, at which the temperature coefficient of frequency (TCF) was close to zero and the turnover temperature was marginally higher than the upper limit of the operating temperature range. The I^2^-BAR structure silicon resonator was suspended with a pair of heating beams to decrease the thermal conductance. The beams also served as electrical leads. The resonator was excited electrostatically and sensed based on piezoresistance. During operation, a voltage was applied across the heating beams to heat the resonator at the turnover temperature. A thermoresistor was included on the chip to monitor the ambient temperature. Fewer leads will result in less power dissipation because the leads are the main routes of thermal dissipation in a vacuum. The temperature of the silicon resonator is not monitored to avoid any additional leads. A lookup-table-based algorithm is used to control the temperature of the resonator. Given that the TCF near the turnover temperature is low, the algorithm is sufficiently accurate, even though the temperature of the resonator is not monitored. The details of the oven-controlled MEMS oscillator can be found in [21,22,23].

Two evaporator boats are included in the micro-evaporator chip to deposit Al to the two far ends of the resonator to facilitate permanent trimming, as shown in Figure 2. The local evaporation trimming has a minor influence on the *Q*-factor because the far ends of the resonators are less stressed, and the stiffness of the resonator is slightly affected. Trace amount of aluminum will not cause a short circuit between the device and electrode because the resonator is released from the substrate and there are oxide layers undercuts under the driving electrodes. Each evaporator boat was covered with a low-stress silicon nitride layer and an aluminum layer on top. The aluminum layer served as the source material for evaporation, whose area and thickness were 20 μm × 38 μm and 0.6 μm, respectively. If 10% of the aluminum layer evaporated on the surface of the resonator, the frequency can be trimmed by ~3346 ppm. The SiN*x* layer serves as the barrier layer, thus preventing aluminum from forming an alloy with the silicon evaporation boat at high temperatures. 

**Figure 3:**
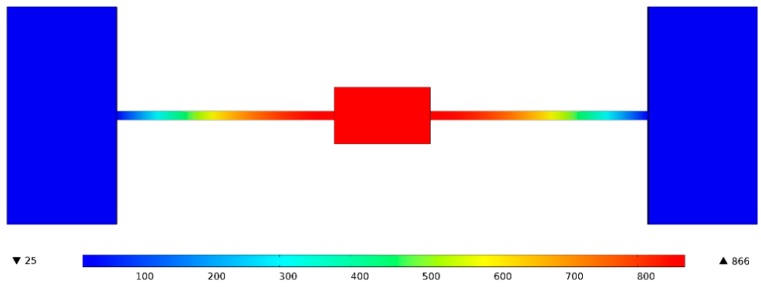
Temperature of the evaporator boat based on simulation is found to be 866 °C when an evaporation power of 108 mW is applied.

Only two hearing beams are used to suspend the evaporation boat, the power consumption for heating can be minimized, and the evaporator boats may be heated at a high temperature with relatively low power owing to their small volumes. Simulation results using COMSOL revealed that the temperature of the evaporator boat may reach 866 °C when 108 mW of evaporation power is applied without considering the heat of fusion and evaporation, as shown in Figure 3. The simulation results are very close to the theoretically calculated results.

## 3. Fabrication

**Figure 4:**
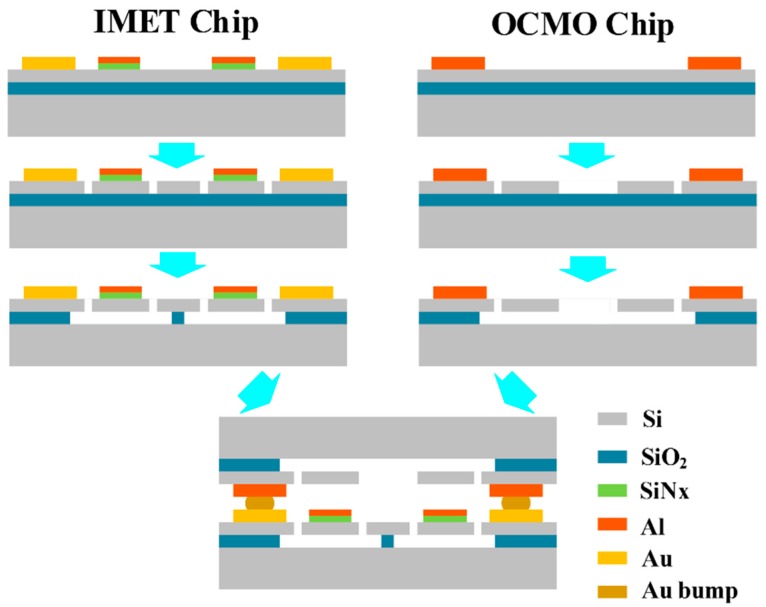
IMET chip and OCMO chip are fabricated separately using SOI MEMS processes and bonded together using a flip-chip machine.

The IMET chip and OCMO chip were fabricated separately with the use of Silicon-on-Insulator (SOI) MEMS processes, and were bonded together with a flip-chip machine, as shown in Figure 4. The processes of the IMET chip are described as follows:

1. The SOI layers are 5 μm thick and heavily boron-doped in the range of 0.001–0.002 Ω·cm. A layer of low-stress silicon nitride with a thickness of 300 nm is deposited using low-pressure chemical vapor deposition (LPCVD) and a layer of 600 nm aluminum is sputtered. Both layers are patterned. The aluminum layer serves as the source material of evaporation and the low-stress silicon nitride serves as the barrier layer.
2. After deposition and patterning of the Cr/Pt/Au electrode, the structures of the micro-evaporators are patterned by deep reactive ion etching (DRIE) and released by HF vapor etching.

The processes used for the silicon resonators are almost the same except that no low-stress silicon nitride and aluminum layers are deposited. The details of the resonator processes can be found in [21,22].

After the Au stud bumps are fabricated on the IMET chip with the wire bonding machine, the IMET chip and the OCMO chip are bonded face-to-face via thermal compression bonding with the flip-chip machine.

**Figure 5:**
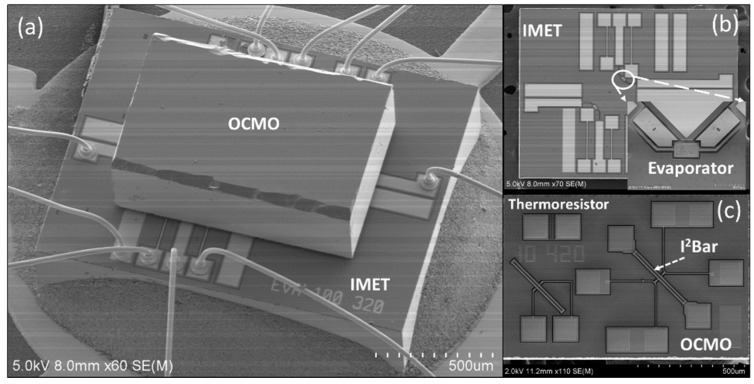
Scanning electron microscopy (SEM) images of the chips: (**a**) device-integrated resonator and micro-evaporator, (**b**) IMET chips and (**c**) OCMO chip.

Figure 5 shows scanning electron microscopy (SEM) images of the chips. The device is then vacuum-packaged in a ceramic carrier. The pressure in the ceramic carrier was estimated to be approximately 500 Pa in other experiments [22].

## 4. Results and Discussion 

### 4.1. Results

**Figure 6:**
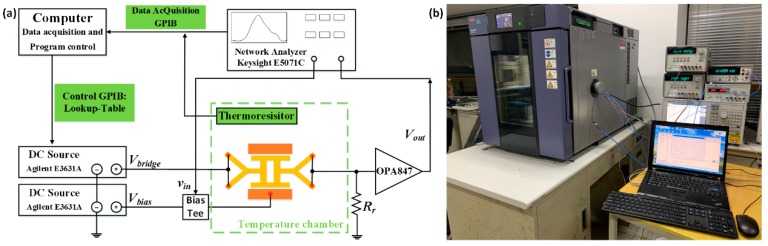
Experimental setup: (**a**) diagram of measurement setup and (**b**) photograph of the actual measurement setup.

The experimental setup used for measuring the output frequency is shown in Figure 6. The resonator is electrostatically actuated and piezoresistively sensed with a discrete resistor *R_r_* connected in series to form a Wheatstone half-bridge. A bridge voltage was applied to the Wheatstone half-bridge to sense and heat the resonator at the turnover temperature, at which the TCF of the resonator is close to zero. The thermoresistor on the chip was used to monitor the change of the temperature in the temperature chamber. The gain of the amplifier on the output of the resonator was approximately 40. The data were transmitted to the computer through the general purpose interface bus (GPIB), and the sampling period is 10 s. 

**Figure 7:**
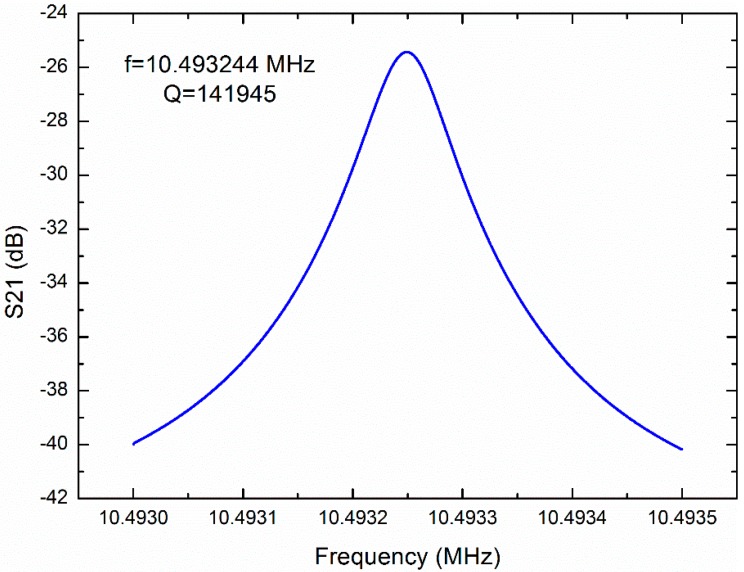
Magnitude–frequency curve measured at 30 °C before trimming. The resonant frequency is measured to be 10.493244 MHz and the *Q*-factor is 141,945.

**Figure 8:**
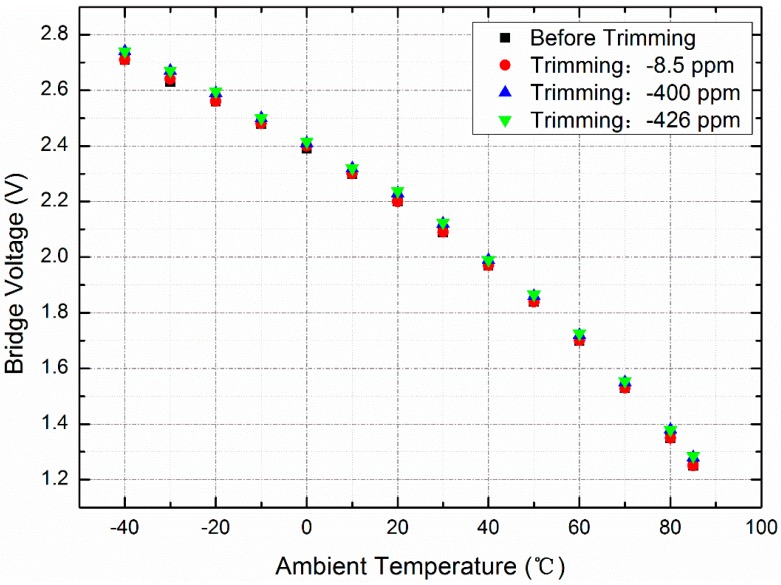
Relationship between the bridge voltage to obtain turnover temperature and the ambient temperature. After a trimming of 426 ppm, the bridge voltage increases by 0.02–0.03 V, but the bridge voltage does not change after a trimming of −8.5 ppm.

**Figure 9:**
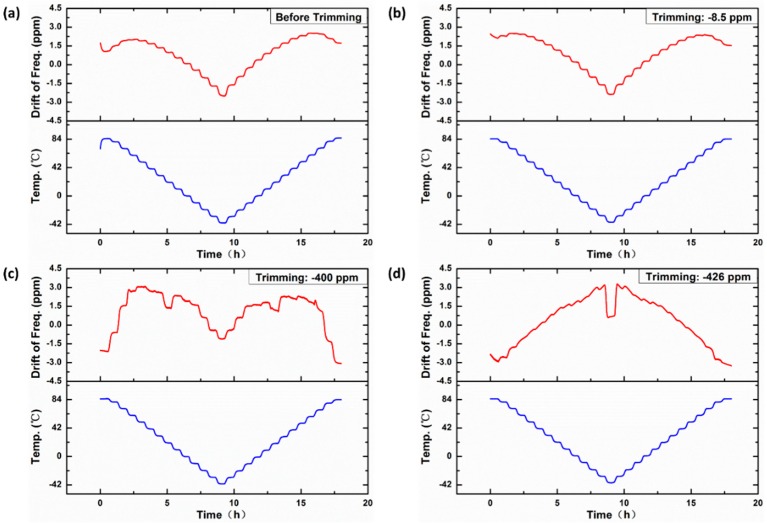
Relationship between the temperature drift of the frequency and ambient temperature over 18 h. (**a**) Before trimming, the temperature drift of the resonant frequency is less than ±2.6 ppm, over the temperature range of −40 to 85 °C. (**b**) The temperature drift drops slightly to ±2.4 ppm after a fine trimming of −8.5 ppm. (**c**) The temperature drift increases slightly to ±3.0 ppm after a coarse trimming of −400 ppm. (**d**) The maximum temperature drift increases to ±3.3 ppm after a trimming of −426 ppm.

Before trimming, the resonant frequency of the device that was vacuum-packaged in a ceramic carrier was measured to be 10.493244 MHz at 30 °C, and the *Q*-factor was 141,945 (Figure 7). The OCMO was characterized in the temperature chamber to obtain the turnover bridge voltage (the bridge voltage heating the resonator to the turnover temperature) at different ambient temperatures, as shown in Figure 8. Use of a piecewise linear fitting method based on the calibration data, a look-up table that contained the turning voltage at any temperature was built. The frequency stability of the resonator was measured with the use of the look-up table in the temperature chamber for 18 h, in which the temperature varies in the industrial temperature range of −40 to 85 °C, as shown in Figure 9a. The temperature drift of the resonant frequency was measured to be less than ±2.6 ppm, over the entire aforementioned temperature range.

**Figure 10:**
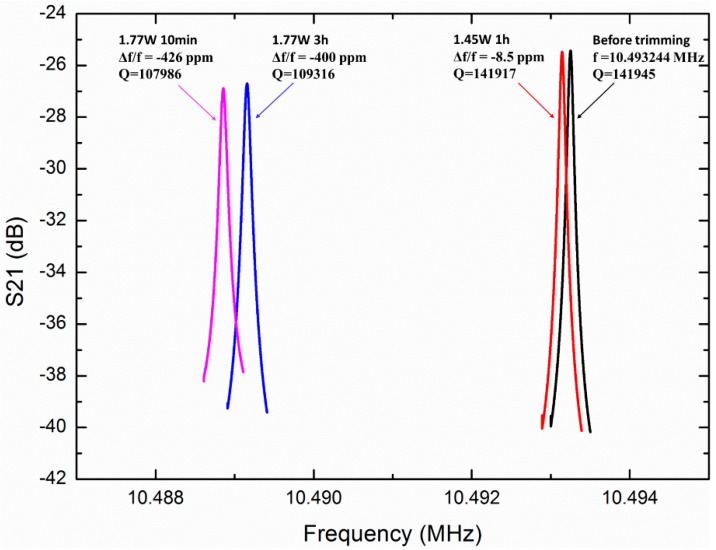
Magnitude–frequency curve of the MEMS resonator, before and after trimming. Before trimming, the resonant frequency is measured to be 10.493244 MHz. The resonant frequency is trimmed to −8.5 ppm with an evaporative power of 1.45 W for 1 h, and trimmed to approximately −400 ppm with an evaporative power of 1.77 W for 3 h, and approximately −426 ppm with an evaporative power of 1.77 W for 10 min.

The resonator is trimmed by the integrated micro-evaporator several times, as shown in Figure 10. The resonant frequency of the resonator is trimmed to approximately −8.5 ppm with a 1.45 W evaporation power for 1 h, and trimmed to approximately −400 ppm with a 1.77 W evaporation power for 3 h and approximately −426 ppm with an evaporative power of 1.77 W for 10 min. The fine- and coarse-trimming for frequency of the OCMO can be achieved by adjusting the evaporation power and time. 

After each trimming, the oscillator is fully characterized to obtain the turnover bridge voltage at different ambient temperatures, as shown in Figure 8, which shows the effect of the trimming on the bridge voltage. After a trimming of 8.5 ppm, no change in the bridge voltage was observed. After a trimming of 426 ppm, the bridge voltage increased by 0.02–0.03 V at different ambient temperatures, which is equivalent to 1.0–2.7%. 

After each trimming, the stability of the resonator is measured by the method described above. After a trimming of −8.5 ppm, the temperature drift of the resonant frequency is less than ±2.4 ppm, as shown in Figure 9b, and it is less than ±3.0 ppm when a coarse trimming of −400 ppm is achieved, as shown in Figure 9c. The maximum temperature drift is less than ±3.3 ppm after −426 ppm trimming, as shown in Figure 9d. The effect of evaporative trimming on frequency stability over the entire temperature range is less than 1.5 ppm. The decrease of frequency stability after trimming is caused by the rise of TCF of the resonator after trimming. Because the TCF of aluminum does not equal to 0 at the turnover temperature, the deposition of aluminum on the resonator surface will inevitably increase TCF of the resonator at the turnover point, which will cause the decrease of frequency stability. 

The *Q*-factor decreases along with trimming slightly, as shown in Figure 10. After −8.5 ppm trimming, the *Q*-factor drops slightly to 141,917 and it drops by 24% to 107,986 after a trimming of −426 ppm. Because the pressure in the ceramic carriers cannot be controlled precisely in our experiments, the *Q*-factors deviate obviously from different devices. Much higher *Q*-factors can be achieved after trimming, as described in the next part. 

**Figure 11:**
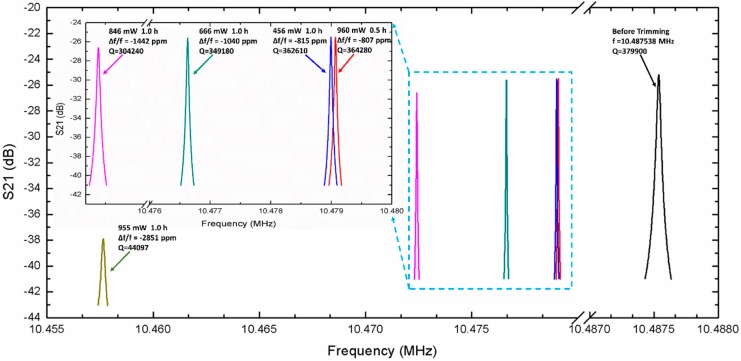
Resonant frequency of the MEMS resonator, before and after trimming. Before trimming, the resonant frequency is measured to be 10.487538 MHz. This frequency is coarse-trimmed at approximately −807 ppm using 960 mW of evaporation power for 0.5 h, and fine-trimmed to approximately −815 ppm using 456 mW of evaporation power for 1 h. The frequency is coarse-trimmed to −1040, −1442 and −2851 ppm, successively, with evaporation powers of 666, 846 and 955 mW, applied respectively for 1 h.

To study the maximum trimming range and the effect of trimming on the *Q*-factor, another device was tested and trimmed in a vacuum chamber, as shown in Figure 11. The resonant frequency of the resonator was measured to be 10.487538 MHz before trimming. This frequency was coarse-trimmed to approximately −807 ppm with an evaporation power of 960 mW for 0.5 h, and fine-trimmed to approximately −815 ppm with an an evaporation power of 456 mW for 1 h. The frequency is coarse-trimmed to −1040 ppm and −1442 ppm successively, with evaporation powers of 666 mW and 846 mW applied respectively for 1 h. The *Q*-factor decreases with trimming, as shown in Figure 11. The *Q*-factor is approximately 379,900 without trimming. After a trimming of −807 ppm, it drops by ~4.1%, to 364,280. It continues to drop by ~0.46% to 362,610 after a trimming of −815 ppm. When the original frequency is trimmed to −1040 and −1442 ppm, it drops to 349,180 and 304,240, respectively, but it is adequately high for a MEMS oscillator, and the value of *f* · *Q* is 3.2 × 10^12^, which is comparable to the bulk mode silicon oscillator reported by others [1]. Finally, the oscillator is further trimmed to −2851 ppm with an evaporation power of 955 mW for 1 h to explore the extreme adjustment ability of IMET. After a trimming of −2851 ppm, the *Q*-factor drops by 88.4% to 44,097. 

**Figure 12:**
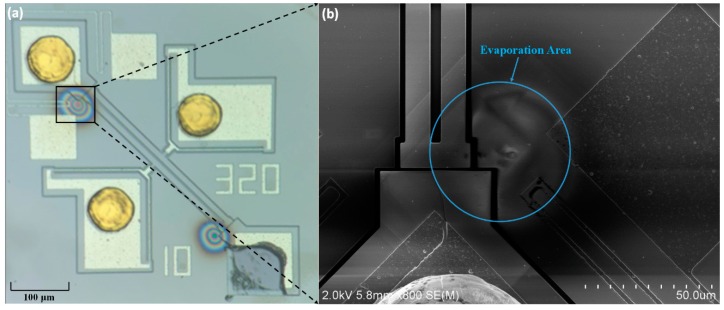
Resonator photograph after trimming: (**a**) optical microscopy image at a magnification of 10×, (**b**) SEM image of the evaporation area.

After trimming, the device was destructed to observe the surface of the resonator. As shown in Figure 12, the color region corresponding to the aluminum evaporation region can be observed on the surface of the resonator. Although the center of the evaporation area deviates from the resonant structure owing to the alignment error of the flip chip welding, the evaporation region covers part of the bar-beam. Because no alignment mark has been made on the chips, the alignment is carried out by means of graphic alignment which introduces a large alignment error (>5 μm). If the alignment is carried out by aligning the marks, theoretically, the alignment error can be controlled at about 1 μm. In addition, if the accuracy of flip chip is improved, the rate of trimming can also be increased. 

### 4.2. Discussion

**Figure 13:**
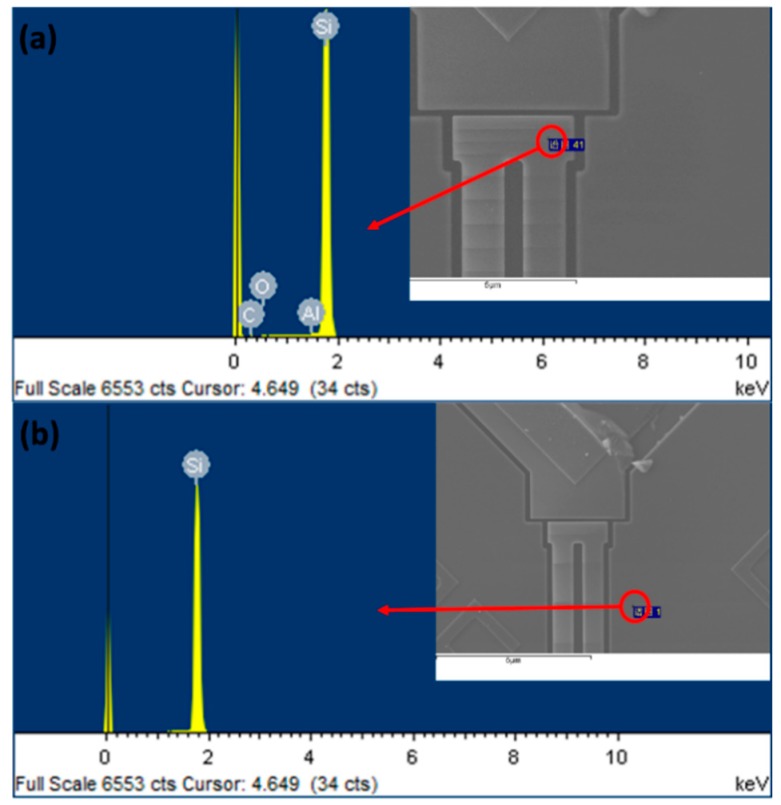
Energy-dispersive X-ray spectroscopic analysis of the content of aluminum: (**a**) the content of aluminum is approximately 0.9% in the evaporation area; (**b**) there is no aluminum in the non-evaporation area.

To verify the existence of aluminum in the evaporation area, an energy-dispersive X-ray spectroscopy (EDX) analysis was employed. As shown in Figure 13a, the evaporation area is characterized and the content of aluminum is approximately 0.9%. For comparison, the non-evaporated area was also characterized. As shown in Figure 13b, no aluminum is present. Because the content of aluminum is so small that it reaches the characterization limit of the EDX, quantitative measurements cannot be performed.

**Figure 14:**
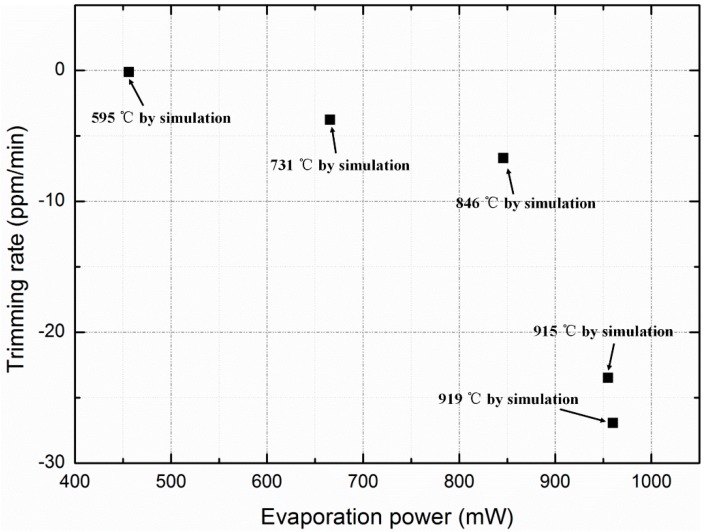
Relationship between trimming power and rate for actual measurements, and the temperature of the evaporation boat simulated by finite element method for each trimming.

Figure 14 shows the relationship between the trimming power and the rate for actual measurements. When the trimming power is less than 846 mW, the power is approximately linearly related with the rate, and the rate increases abruptly when the power is higher than 846 mW. The actual trimming rate measured in the experiment is much lower than the theoretical value estimated by Equations (2)–(4) owing to several reasons. Firstly, because the micro-evaporator is bonded to the resonator through Au bumps which are more than 40 μm high, the evaporation coefficient α is much lower than one. In the theoretical estimation, the temperature drop of aluminum source caused by heat dissipation during evaporation is not considered, and the effect of dense native oxide layer on the evaporation rate of aluminum source is not considered. As a matter of fact, aluminum is very susceptible to oxidation at high temperature in the sealed cavity with pressure of 500 Pa. The dense oxide layer might play a significant role in decreasing the measured deposition rate, which is much lower than the theoretical results as shown in Figure 14. In addition, because the HF vapor corrodes the chromium adhesion layer of the electrode pad in our lab, the buried oxide under the evaporation boat is not released. Accordingly, this greatly increases the heat dissipation through the substrate.

According to the heating power measured, the temperature of the evaporation boat is simulated by finite element method, as shown in Figure 14. Compared with the theoretical results in Figure 1, the simulated temperature is much higher than the theoretical temperature at the same evaporation rate, which is caused by the reasons analyzed above, including the heat dissipation during evaporation, oxidation of aluminum and the misalignment issue.

## 5. Conclusions

This study reports an IMET technology to permanently trim the frequency of an OCMO. Because the various parts of the MEMS oscillator are extremely small, the resonant frequencies can be trimmed significantly with trace amounts of material deposition. Theoretically, 36.8 pg of deposited material can achieve approximately a permanent frequency trimming of −1000 ppm. The micro-evaporators suspended by heating beams were designed and heated electrically, and the aluminum layers on the surface of the evaporator were evaporated and deposited on the surface of the resonator. When the evaporator was heated at 850 °C, the resonant frequency could be theoretically trimmed by more than −1000 ppm within 2 min in vaccum. The micro-evaporators were bonded to the OCMO integrated face-to-face and encapsulated in vacuum. During trimming, the micro-evaporators were heated electrically, and the aluminum layer on the surface was evaporated and deposited on the surface of the resonator that trimmed the resonant frequency of the oscillator permanently. The effect of IMET trimming on the frequency stability of OCMO was minor. The temperature drift of the resonant frequency was measured to be less than ±2.6 ppm over the ambient temperature range from −40 to 85 °C without trimming, and the maximum temperature drift increased slightly to ±3.3 ppm after a permanent trimming of −426 ppm. The trimming rate can be controlled by the electric power of evaporation. A resonator was coarse-trimmed by approximately −807 ppm with the use of an evaporation power of 960 mW for 0.5 h, and was fine-trimmed by approximately −815 ppm with the use of an evaporation power of 456 mW for 1 h. Even though the *Q*-factor decreased after trimming, a *Q*-factor of 304,240 was achieved after a trimming of −1442 ppm which is adequately high for the oscillator. An energy-dispersive X-ray spectroscopic analysis was employed to verify the existence of aluminum in the evaporation area. It is demonstrated that the OCMO with IMET can achieve the required frequency stability over the industrial temperature range and the necessary permanent frequency trimming after vacuum packaging.
